# Novel treatments for congenital adrenal hyperplasia

**DOI:** 10.1007/s11154-022-09717-w

**Published:** 2022-02-23

**Authors:** Mariska A. M. Schröder, Hedi L. Claahsen - van der Grinten

**Affiliations:** 1 Department of Pediatrics, Amalia Childrens Hospital, Radboud University Medical Center, Nijmegen, The Netherlands; 2 Department of Laboratory Medicine, Radboud Institute for Molecular Life Sciences (RIMLS), Radboud University Medical Center, Nijmegen, The Netherlands

**Keywords:** Congenital Adrenal Hyperplasia, 21-hydroxylase deficiency, Glucocorticoid replacement therapy, Modified-release, Corticotropin-releasing hormone receptor antagonist, Adrenocorticotropic hormone antagonist, Steroid production inhibitors

## Abstract

Patients with classic congenital adrenal hyperplasia due to 21-hydroxylase deficiency (21OHD) need life-long medical treatment to replace the lacking glucocorticoids and potentially lacking mineralocorticoids and to lower elevated adrenal androgens. Long-term complications are common, including gonadal dysfunction, infertility, and cardiovascular and metabolic co-morbidity with reduced quality of life. These complications can be attributed to the exposure of supraphysiological dosages of glucocorticoids and the longstanding exposure to elevated adrenal androgens. Development of novel therapies is necessary to address the chronic glucocorticoid overexposure, lack of circadian rhythm in glucocorticoid replacement, and inefficient glucocorticoid delivery with concomitant periods of hyperandrogenism. In this review we aim to give an overview about the current treatment regimens and its limitations and describe novel therapies especially evaluated for 21OHD patients.

## Background

Congenital adrenal hyperplasia (CAH) comprises of a group of autosomal recessive disorders caused by mutations in one of the genes encoding enzymes that are involved in the adrenal steroidogenesis [1, 2]. In more than 95% of all cases, CAH is due to mutations in CYP21A2, leading to 21-hydroxylase deficiency (21OHD). In this paper we solely focus on 21OHD. This enzymatic defect leads to a decreased production of cortisol with consequently lack of the negative feedback towards CRH and ACTH secretion from the hypothalamus and anterior pituitary, respectively. Chronically elevated ACTH levels lead to chronic stimulation of the adrenal cortex with accumulation of adrenal precursor steroids before the enzymatic block, that in turn are shifted into the non-affected adrenal androgen pathway. Therefore, typical biochemical characteristics of 21OHD are a lack of cortisol and in the most severe cases also a lack of aldosterone, with increased concentrations of adrenal androgens and precursor steroids. The adrenal precursor steroids 17-hydroxyprogesterone (17OHP), androstenedione, and 21-deoxycortisol are typically elevated in 21OHD and can be quantified by mass spectrometry to confirm the enzymatic defect and to monitor treatment [3]. Next to these traditional biomarkers, 11-oxygenated androgens may have potential in the management of CAH but are not yet routinely measured [4, 5]. Steroid biomarkers are commonly measured in blood or saliva, and their metabolites can also be measured in urine [6]. In many countries around the world, measurement of 17OHP is added to the neonatal screening to detect classic CAH already in early life to prevent severe salt wasting crisis just after birth [7, 8].

The clinical presentation of 21OHD depends on the residual enzymatic activity. Neonates with the most severe form of 21OHD, the classic salt wasting form, have no residual enzymatic activity [9] and present signs of salt wasting and dehydration already in the first weeks of life. Girls are variably virilized due to the elevated androgen exposure already in utero [1]. A residual enzymatic activity of 1 – 2% is generally enough to produce sufficient amounts of aldosterone to prevent life threatening salt wasting and is referred to as the classic simple virilizing form. These children still suffer from insufficient cortisol production and strongly elevated androgens resulting in a diversity of symptoms such as increased height velocity, signs of precocious puberty and advanced bone maturation [10]. A residual enzymatic activity of 30 – 50% leads to the mildest form of 21OHD, the non-classic 21OHD. Most patients still have some suboptimal cortisol production and subsequently slightly elevated ACTH production. Symptoms of premature pubarche, irregular menstruation, acne, hirsutism can be attributed to the slightly elevated adrenal androgens in these patients [11, 12].

## General treatment goals

21OHD treatment firstly aims to substitute the deficient steroid hormones, thereby preventing the occurrence of adrenal and salt-wasting crises, and secondly to restore the negative feedback mechanism towards hypothalamic CRH and pituitary ACTH secretion to diminish chronical stimulation of the adrenal cortex with consequently adrenal androgen overproduction [2]. The prevention of Addisonian crisis follows general recommendations of stress dosing and education. Patients should be trained in increasing their glucocorticoid dosages properly in situations of illness. Ideally, glucocorticoid substitution should replicate the normal physiological rhythm of cortisol as close as possible [13]. Normally, ACTH and cortisol levels follow a circadian rhythm with nadir levels at night, which start to rise between 2 and 4 AM, peak shortly after waking, and gradually decline during the day [14]. Consequently, in 21OHD patients the production of adrenal androgens is highest in the early morning. In contrast to other forms of adrenal insufficiency, in order to efficiently suppress the adrenal androgen production, often supraphysiological doses of glucocorticoids are required to restore the negative feedback loop [15]. Treatment with supraphysiological dosages of glucocorticoids is associated with the risk of unfavorable effects similar to hypercortisolism, such as weight gain, cushingoid features and metabolic syndrome [16, 17]. On the other hand, undertreatment entails the risk to develop a life threatening Addisonian crisis [18] and signs of hyperandrogenism. There is little consensus on how to address the adrenal androgen overproduction. For individual patients, a reverse circadian treatment pattern, with the highest glucocorticoid dose in the evening, can be recommended to better suppress the early morning rise in ACTH and consequently adrenal androgens [19]. Unfortunately, a good balance between over- and undertreatment is still a challenging task, and patients with 21OHD may simultaneously be over- and undertreated at different time points of the day. Long-term complications in 21OHD include gonadal dysfunction, infertility, and cardiovascular and metabolic co-morbidity with reduced quality of life (QoL) [20–22]. Frequent and adequate monitoring of treatment is recommended to minimize these long-term risks.

## Conventional treatment in different age groups

Treatment strategies differ between age groups [23]. In childhood, the primary treatment goals besides the prevention of Addisonian crisis are achievement of normal growth and weight to reach a final height within the target range, and a normal timing of puberty. Suboptimal suppression of adrenal androgens, with subsequent aromatization to estrogens, can lead to advancement of bone age with consequently decreased final height. Furthermore, signs of precocious puberty can develop already at an early age. For children and adolescents, hydrocortisone (HC) -generally given as tablets or suspensions [24]- is the preferred glucocorticoid formulation, because of its short half-life of 1–2 h. For this reason, HC is generally dosed in 3 different dosages over the day. The recommended substitution dose of HC in patients with adrenal insufficiency is generally 8 – 12 mg/m2/day with increased dosages in case of illness or surgery [2, 25], but for 21OHD patients often higher dosages are necessary to suppress adrenal androgens sufficiently [17]. Importantly, there is a great variability in HC sensitivity between patients, and therefore HC dosing must be monitored carefully by frequently measuring 17OHP and androstenedione levels, preferably at different time points over the day to individualize treatment. The use of long acting more potent glucocorticoids should be avoided as these preparations can lead to growth suppression and features of hypercortisolism [26].

In adolescents, normal final height and normal gonadal function are the main goals of treatment. During puberty, an increased clearance of glucocorticoids is observed due to a decreased activity of 11-β hydroxysteroid dehydrogenase explaining the need of higher glucocorticoid dosages in this period [27]. However, increased dosages of glucocorticoids are negatively correlated with reduced final height and, therefore, dosages above 17 mg/m2/day should be avoided. Poor hormonal control is related to gonadal dysfunction, already in adolescents. In males with classic CAH and poor hormonal control, development of testicular adrenal rest tumors (TART) can already occur in early puberty or even during childhood [28]. In girls, poor hormonal control can cause menstrual disturbances [29]. An additional challenge, especially for this age group, is the lack of therapy compliance leading to poor hormonal control with increased morbidity and mortality [30].

After reaching final height the main aim of treatment is to prevent infertility, co-morbidity, and optimize QoL [31]. In adult patients, also longer acting glucocorticoids such as dexamethasone or prednisone are used for specific indications such as the improvement of fertility [24]. One important advantage is the possibility to dose these preparations only twice a day, thereby improving compliance. The lowest dose of glucocorticoids that is necessary to suppress adrenal androgens should be used.

Cortisone acetate is not recommended in the treatment of 21OHD, because of its lower availability compared to HC and the need to be converted to cortisol by 11-beta hydroxysteroid dehydrogenase [32].

## Limitations of current therapy

Inadequate hormonal control and long-term adverse health outcomes, including obesity, hypertension, metabolic syndrome, reduced final height, subfertility, hirsutism in women, and development of TART in men, are very commonly observed in CAH patients with current treatment strategies despite efforts from patient and health professionals [16, 20], necessitating the development of new therapeutic strategies. The current glucocorticoid substitution therapy that is established for patients with all forms of adrenal insufficiency has important limitations for CAH patients; conventional immediate-release glucocorticoid preparations fail to effectively suppress the HPA-axis that is necessary to suppress adrenal androgen production. Immediate-release formulations result in high glucocorticoid levels after each dose, that are potentially followed by a period of low levels of glucocorticoids after 4 – 6 h [33–35], leading to periods of inefficient suppression of the HPA-axis and the need of higher glucocorticoids to achieve this goal.

## Novel therapy

**Fig. 1:**
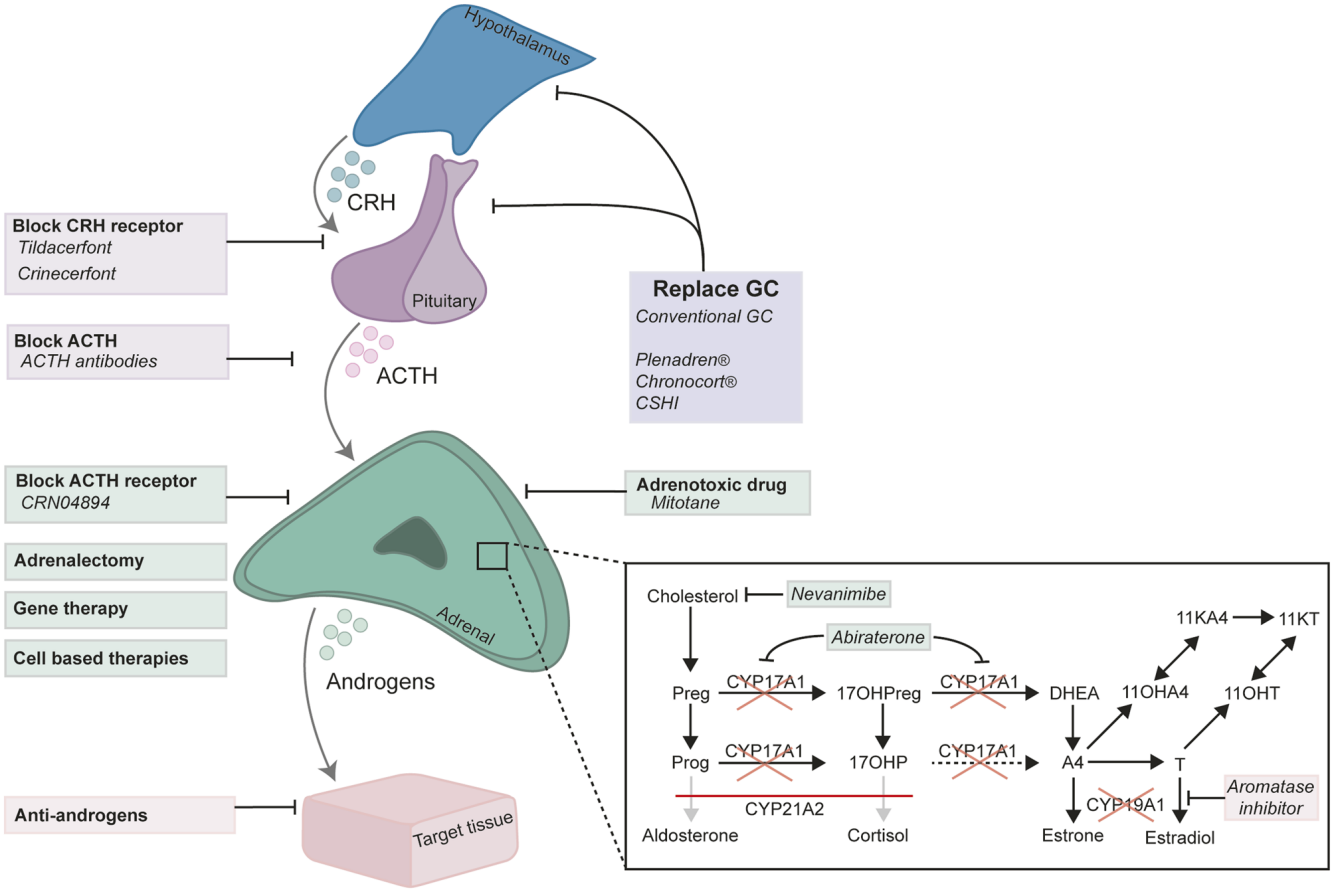
Novel therapeutic approaches for patients with 21OHD include the replacement of glucocorticoids (GC) by improved modified-release HC preparations (Plenadren^®^, Chronocort^®^) or improved modes of GC delivery (CSHI), associated with more efficient suppression of hypothalamic–pituitary–adrenal (HPA)-axis driven adrenal androgen production. This HPA-axis-driven adrenal androgen production may be additionally blocked by corticotropin releasing hormone (CRH) receptor blocking (Tildacerfont, crinecerfont) or ACTH (receptor) blocking. Alternatively, adrenal androgen production may be inhibited by adrenal steroidogenesis blockers (Nevanimibe, abiraterone acetetate), adrenotoxic drugs (mitotane), or bilateral adrenalectomy. Inhibition of the aromatization of adrenal androgens may lower the suppressing effects of estrogens on growth and (co)-administration of anti-androgens may inhibit hyperandrogenic effects. Gene and cell-based therapies anticipate restoring the HPA-axis regulated glucocorticoid and mineralocorticoid production

In recent years new therapeutic approaches have emerged (Fig. 1, summarized in Table 1) that improve dosing or better mimic the physiological glucocorticoid levels and thereby more effectively suppress the high ACTH and androgen production in the early morning (modified-release glucocorticoid preparations and continuous subcutaneous glucocorticoid infusion). In addition, complementation of glucocorticoid treatment with novel non-glucocorticoid treatment approaches interfering with the HPA-axis to diminish the effects of CRH or ACTH, inhibit adrenal steroidogenesis, or antagonize the effects of adrenal androgens are promising therapeutic strategies to adapt or lower the glucocorticoids to physiological levels. While above mentioned additional therapeutic approaches still require daily administration of medicines, future gene and cell-based therapeutic approaches anticipate curing the enzymatic defect and thereby restoring the HPA-axis-regulated glucocorticoid and mineralocorticoid production.

**Table 1:** Overview of (future) therapeutic approaches with their indications and benefits and limitations

**Approach**	**Medication**	**Indications and benefits**	**Limitations**	**Ref**
	***(Status)***			
Improved glucocorticoid preparations and administration				
**Low dose HC granules and tablets**	Alkindi^®^ (Licensed)	- Infants and children with CAH.	- Short half-life may result in periods of undertreatment.	[35, 37–39]
		- Allows accurate dosing (0.5, 1, 2, 5 mg).	- No effective suppression of early morning ACTH surge.	
		- Taste-masking.	- Does not replicate physiological cortisol rhythm.	
			- Expensive.	
			- Not available in all countries.	
**Dual release**	Plenadren^®^ (Licensed)	- Not recommended for CAH.	- No effective suppression of early morning ACTH surge.	[40, 41]
		- Once-daily dosing.		
		- Mimics physiological rhythm of cortisol.		
		- Safe and well-tolerated.		
**Modified release **	Chronocort^®^ (Licensed)	- Children above 12 years of age and adults with CAH.	- Data on long-term efficacy and effect on prevalence of long-term complications not yet available.	[14, 45–47]
		- Safe and well-tolerated.	- The potential combination of Chronocort^®^ (evening) and immediate-release HC (morning) has not been studied.	
		- Does more effectively suppress early morning surge of ACTH.	- Requirement of immediate-release GC for stress dosing.	
		- Improves biochemical control despite lower total GC dose.	- Not available for children.	
		- Approximates physiological cortisol rhythm.		
		- Allows for twice-daily ‘toothbrush’ regimen.		
		- Improved sperm count in men with TART reported.		
		- Restoration of menses reported.		
**Continuous subcutaneous infusion **	Hydrocortisone (Licensed)	- Elevated androgens and/or supraphysiological dosages of HC. - Especially for 21OHD patients with lowered HC bioavailability and/or rapid clearance.	- Invasive procedure, impractical, local skin reactions, expensive, constant reminder of illness.	[49–55, 57]
		- Replicates physiological cortisol rhythm.	- Preference for CSHI or improved QoL not evident from all studies with patients with AI.	
		- Improves biochemical control despite lower total GC dose.		
		- Possibility to individualize delivery rates.		
		- Potential for replication of pulsatile ultradian rhythm.		
		- Restoration menses and reduction TART reported.		
		- Compliance can be monitored.		
Interference with HPA axis - lowering ACTH production or ACTH effect				
**CRF1 receptor antagonists **	Tildacerfont (Two phase 2b ongoing)	- Elevated androgens and/or supraphysiological dosages of HC.	- Additional drug administration.	[60]
		- Safe and well-tolerated.	- Drug-drug interaction with dexamethasone.	
		- Biochemical control improves in poorly controlled patients	- Not all patients respond to treatment.	
		- Reduction of TART reported.	- Long-term studies are not yet available.	
		- May allow GC dose reduction (in well-controlled patients).		
	Crinecerfont (Two phase 3 ongoing)	- Elevated androgens and/or supraphysiological dosages of HC.	- Additional drug administration.	[61]
		- No serious adverse events reported so far.	- Long-term studies are not yet available.	
		- Dose-dependent reduction of ACTH, 17OHP, and androstenedione.		
		- May allow GC dose reduction (in well-controlled patients).		
		- Elevated testosterone levels in men.		
**ACTH antibodies **	ALD1613 (Preclinical)	- Elevated androgens and/or supraphysiological dosages of HC.	- Additional drug administration.	[62, 63]
	ALD1611 (Preclinical)	-Reduces circulating glucocorticoid levels in rodents and monkeys.	- Not yet studied in clinical trial.	
**ACTH receptor (MC2R) antagonist**	CRN04894 (Phase 1 ongoing)	- Elevated androgens and/or supraphysiological dosages of HC	- Additional drug administration.	[65]
		- Preliminary non-peer reviewed results suggest clinically relevant reduction in cortisol in healthy individuals.	- Only preliminary non-peer reviewed data currently available.	
Targeting adrenal steroid production				
**Selective ACAT1 blocker**	Nevanimibe (stopped)		- Side effects are common.	[68]
			- Not effective in all patients.	
**17-20 lyase inhibitor**	Abiraterone acetate (Phase 1 finished, phase 1 ongoing, phase 2 planned)	- Elevated androgens and/or supraphysiological dosages of HC.	- Not selective to 17,20-lyase.	[71, 72]
		- No severe adverse events.	- Lowering of GC dose is potentially associated with elevated ACTH levels. Long-term use may predispose to TART development.	
		- Decreases adrenal androgens.	- May affect gonadal steroidogenesis.	
		- May allow GC dose reduction.	- Not suitable for long-term treatment.	
**Selective adrenotoxic drugs**	Mitotane (Licensed)	- To restore fertility in men with TART; only when TART did not respond to increasing GC doses; when semen cryopreservation was not done or not successful.	- Potential teratogenicity, significant toxicities.	[76–78]
			- Drug-drug interactions with hydrocortisone, requiring higher HC dose.	
			- No effective TART reduction in all men.	
**Adrenalectomy**		- Not recommended	- Increased risk to develop Addisonian crisis, Nelsons syndrome, TART, or ovarian adrenal rest tumors.	[79–81]
Targeting androgen function				
**Aromatase inhibitors to inhibit the conversion of androgens to estrogens**	Anastrozol (Licensed)	- Decelerate bone maturation in children with predicted low adult height in comparison to target height and severely advanced bone age.	- No consensus on how to monitor disease control biochemically.	[83, 84]
	Arimidex (Licensed)			
	Aromasin (Licensed)			
	Letrozole (Licensed)			
**Combination of anti-androgen with aromatase inhibitor**	Flutamide (Licensed)	- Children with advanced bone age and small predicted adult height; to improve linear growth and suppress bone maturation.	- Reduced HC dosing results in elevated ACTH, potentially predisposing to TART development.	[85–88]
	Testolactone (Licensed)	- Allows for lower physiological HC doses.	- Risk for hepatotoxicity.	
	(Phase 2 ongoing)		- Flutamide affects clearance HC.	
			- Long-term data on efficacy still lacking.	
Restore HPA-axis regulated glucocorticoid and mineralocorticoid production				
**Cell therapy**	Preclinical	- All patients with 21OHD.	- Limited *in vivo* studies with adrenal-like steroidogenic cells.	[95–98]
		- No need for daily GC replacement.	- Not yet studied in clinical trial.	
		- Steroid production could be adapted to physiological demand.		
**Gene therapy**	Phase 1	- All patients with 21OHD.	- Therapy duration may be limited.	[89, 90, 93, 94]
		- No need for daily GC replacement.		
		- Steroid production could be adapted to physiological demand.		

### Improved glucocorticoid preparations

Although HC is currently the preferred preparation for the treatment of children and adults with adrenal insufficiency, the availability of licensed preparations is limited. Generally, HC tablets of 5, 10, and 20 mg are available, but these dosages are not useful in young children with the lowest recommended dosages of up to 0.5 – 1 mg. Therefore, traditionally HC capsules based on crushed tablets are often used but the variability in concentrations often exceeds 25% [36]. To improve glucocorticoid treatment in young infants and children, HC granules were developed in dosages of 0.5, 1, 2, and 5 mg (Alkindi^®^, Diurnal Europe B.V.) [37, 38]. A 2-year prospective study showed that these HC granules are accurate in dosing without signs of over- or undertreatment and without increased risk to develop Addisonian crisis [39]. These tablets are now licensed and available in Europe and in the United States. In the Netherlands, HC tablets of 1, 5, and 10 mg are available as ACECORT tablets (ACE Pharmaceuticals B.V.) for pediatric and adult patients with all forms of adrenal insufficiency.

Two modified-release HC (MR-HC) formulations have been developed; a dual-release formulation (Plenadren^®^) and a delayed and sustained formulation (Chronocort^®^).

Plenadren^®^ (Shire Pharmaceuticals Ireland Limited) is a once-daily sustained release HC formulation with an extended release core and an immediate release coating and is licensed for the treatment of adults with adrenal insufficiency with the aim to improve physiological patterns. Peak cortisol levels are observed within 50 min after administration [40]. While this formulation allows for once daily administration and better mimics the physiological cortisol rhythm than conventional glucocorticoid therapy in patients with adrenal insufficiency with significant positive effects on metabolic control, Plenadren^®^ is not considered suitable for 21OHD patients, as a once-daily morning administration does not suppress the early morning surge of ACTH and adrenal androgens in 21OHD, obeying elevated androgens at this period of the day [41].

Chronocort^®^ (Diurnal, UK), a modified-release HC formulation, is a more promising glucocorticoid formulation for 21OHD patients, because it aims to address the overnight rise of ACTH and adrenal androgens. Chronocort^®^ consists of an inert core onto which HC is coated, covered with a pH-sensitive delayed release coat. Based on pharmacokinetic modeling, physiological cortisol levels in adults could be best achieved by a dose combination of 15 or 20 mg Chronocort^®^ at 11 PM and a smaller dose of 10 mg Chronocort^®^ at 7 AM [14]. As for the administration of immediate-release HC [42, 43], taking Chronocort^®^ with food delays and reduces the cortisol peak [44]. Chronocort^®^ treatment normalized 17OHP and androstenedione levels [45] and significantly reduced excretion of urinary 17OHP and androgen metabolites compared to baseline conventional therapy [46], despite lowering the total daily glucocorticoid dose [45]. QoL and fatigue scores at baseline were similar to a healthy population and did not improve upon six-month Chronocort^®^ treatment [45]. Greater improvements were observed for patients on Chronocort^®^ versus conventional treatment, with patients on Chronocort^®^ having superior biochemical control in the morning and afternoon [47]. One male patient with a history of TART had improved sperm count during Chronocort^®^ treatment. A phase 3 study is planned to compare the efficacy, safety, and tolerability of twice-daily Chronocort^®^ versus twice-daily immediate-release HC in 21OHD patients of 16 years and older (NCT05063994). It is of interest to evaluate whether the improved disease control and more effective suppression of ACTH and adrenal androgens by Chronocort^®^ treatment translates into lower prevalence of long-term complications.

### Improved glucocorticoid administration

Continuous intravenous or subcutaneous HC infusion via a programmable pump more closely replicates the normal circadian rhythm of cortisol compared to conventional therapy in patients with adrenal insufficiency [48–54]. In 21OHD, continuous HC infusion lowers morning ACTH and 17OHP levels to near normal [48]. Prolonged continuous subcutaneous HC infusion (CSHI) is found to be safe and well-tolerated [49–51, 54]. CSHI can especially be useful for patients with abnormal HC pharmacokinetics, i.e. lowered HC bioavailability and/or increased HC clearance [49, 51, 54]. Disease control improved despite lowering total daily HC dose [49, 51]. Yet, improved QoL has not been reported by every study assessing CSHI for patients with adrenal insufficiency. In a double-blinded placebo-controlled trial assessing QoL, only five out of ten patients with Addison’s disease blindly preferred CSHI with placebo control administration over placebo infusion with conventional oral HC intake, while four patients preferred conventional HC intake and 1 patient was uncertain [52]. Nonetheless, a six months open-label phase 1/2 study completed by eight adults with difficult-to-treat CAH showed improved health-related QoL [55]. Biochemical control, including levels of 11-oxygenated androgens [56], and sleep also improved by CSHI in these CAH patients [55]. While a programmed earlier increase of cortisol better suppressed morning ACTH and 17OHP in two patients, this was not tolerated as it led to early-morning awakenings [55, 57]. Interestingly, testosterone was lowered by CSHI in women but increased in men by CSHI suggesting an improvement of the testicular function due to improved disease control[56]. Six of the eight patients expressed the desire to continue with CSHI, mainly because of improved health related QoL [55]. Further follow-up of five of these patients continuing using CSHI showed that the observed improvements in disease control and health related QoL maintained for another year [57]. In one of four reported patients with CAH and bilateral TART that were treated with CSHI, TART size reduced by 40% after CSHI [50, 55].

Important limitations of CSHI are the invasive procedure, local skin reaction at the catheter site [53, 55, 57], and financial constraints [57].

### Interference with hypothalamic–pituitary–adrenal axis

Novel non-glucocorticoid treatment approaches to block the HPA-axis have emerged as ACTH is the major stimulation of the adrenal cortex. These drugs still require co-administration of (novel formulations of) glucocorticoids to replace cortisol. This ‘block and replace’ concept [58, 59] aims to suppress the HPA-axis more efficiently, which may allow further reduction in glucocorticoid doses.

#### Targeting the pituitary gland

Corticotropin-releasing factor (CRF), a.k.a. corticotropin-releasing hormone (CRH), is the main regulator of pituitary ACTH synthesis and secretion, where it binds to the CRF type 1 (CRF1) and CRF type 2 (CRF2) receptors. Antagonizing the CRF receptor has the potential to lower the ACTH production and concomitant androgen excess in CAH, without the adverse effects of supraphysiological glucocorticoid doses. Few CRF1 antagonists are being evaluated for safety and efficacy in 21OHD patients.

The CRF1 antagonist Tildacerfont (Spruce Bioscience, USA) was evaluated for pharmacokinetic parameters, safety and efficacy in adults with 21OHD in two phase 2 studies [60]. During the first study, patients received either escalating doses (200-1000 mg) of Tildacerfont once-daily (n = 10) or received 100 or 200 mg of Tildacerfont twice daily (n = 16). During the second study, patients received 400 mg once daily for twelve weeks (n = 11). Tildacerfont was generally well tolerated and safe, and peak levels were observed about 5–6 h after administration. Importantly, both dexamethasone and Tildacerfont are primarily metabolized by CYP3A4, resulting in an approximate two-fold increased dexamethasone exposure when given concomitantly. Therefore, participants receiving dexamethasone were excluded from efficacy analysis. Additional Tildacerfont administration improved ACTH, androstenedione, and 17OHP levels in participants with poor disease control at baseline (based on androstenedione being more than 2 × the upper limit of normal), but did not reduce 17OHP and androstenedione levels in a clinically relevant manner in participants with good disease control. One poorly controlled man did not respond to treatment. Corresponding to the observed changes in ACTH levels, TART volume reduced in one man (23%) or visually regressed in one man of a total of three men with TART and post-treatment ultrasound. However, one of the two patients demonstrating TART reduction was treated with dexamethasone, and thus the regression of TART in this patient may also be due to the increased exposure to dexamethasone. Future larger studies are ongoing to unravel which patients exhibit poor response to Tildacerfont. Prolonged efficacy and safety of Tildacerfont administration in poorly controlled patients, and the potential of Tildacerfont treatment to reduce glucocorticoid dose in well controlled CAH patients are currently being evaluated in long-term multicenter placebo-controlled trials (NCT04457336; NCT04544410).

Crinecerfont (NBI-74788; Neurocrine Biosciences, Inc, USA) is another potent CRF1 receptor antagonist that is currently under evaluation for children (phase 2, NCT04045145; phase 3, NCT04806451) and adults (phase 3, NCT0449091) with 21OHD. A phase 2 clinical trial -including 7 male and 11 female 21OHD patients with inadequate disease control- evaluated the safety, tolerability, and efficacy of four Crinecerfont regimens, each dosed for 14 days while continuing normal glucocorticoid treatment [61]. Crinecerfont treatment was well-tolerated and led to a dose-dependent decrease in ACTH levels (median change of 54%-66% in the morning (6 AM – 10 AM) depending on regimen) and consequent reductions in 17OHP (53%-64%) and androstenedione (21%-64%) levels relative to baseline. No serious adverse events occurred. Crinecerfont also reduced testosterone levels in females and reduced the androstenedione/testosterone ratio in men from baseline. Due to the small sample size and variable adrenal steroid levels at baseline, no statistically significant effect of treatment, nor differences between regimens could be observed [61]. Nevertheless, because of the consistent decline of both 24-h and morning (6 AM – 10 AM) levels of ACTH, 17OHP, and androstenedione, Crinecerfont holds great promise in decreasing androgen levels and potentially allows for reducing glucocorticoid doses. The extent to which the daily glucocorticoid dose could be reduced by the additional administration of Crinecerfont, as well as the tolerability, efficacy, and safety of chronic Crinecerfont use is currently being evaluated in the phase 3 clinical trial.

#### Targeting ACTH function

The negative effects of ACTH could potentially be diminished by ACTH antagonists [62, 63] or ACTH receptor (MC2R) blocking [58, 64]. Two preclinical studies tested the efficacy of an ACTH-neutralizing antibody (ALD1613 or ALD1611) in cell lines, rodents, and monkeys [62, 63]. The antibody ALD1613 reduced ACTH-induced MC2R signaling [62] and both antibodies were reported to reduce circulating glucocorticoid levels [62, 63]. Alternatively, not ACTH, but its receptor MC2R could be targeted by a receptor antagonist. Antagonizing MC2R can dose-dependently lower cortisol levels in primary cultured ACTH-induced canine adrenocortical cells [64]. Currently, a double-blind, randomized, placebo-controlled phase 1 study is assessing the safety, tolerability and efficacy (steroid hormone suppression following ACTH stimulation) of an oral ACTH antagonist CRN04894 (Crinetics Pharmaceuticals) in healthy individuals. Preliminary non-peer reviewed results of this trial suggest that CRN04894 was well-tolerated in 39 enrolled healthy individuals with a clinically relevant reduction in cortisol [65]. Clinical studies need to follow to study the efficacy and safety of ACTH and MC2R antagonists in CAH patients and their potency to lower glucocorticoid doses in CAH while providing or improving biochemical control.

#### Targeting steroid production

Two adrenal steroidogenesis blockers have been clinically tested in patients with 21OHD to lower the production of adrenal precursor steroids and adrenal androgens despite persistently elevated ACTH: Nevanimibe and Abiraterone acetate.

Nevanimibe (ATR-101; Millendo Therapeutics, Ann Arbor, MI, USA) is a potent and selective inhibitor of acyl-coenzyme A: cholesterol O-acyltransferase 1 (ACAT1). ACAT1 catalyzes the conversion of free cholesterol to cholesteryl esters, which can be stored in adrenocortical cells for steroid hormone biosynthesis. Inhibition of ACAT1 decreases the cholesterol ester availability for adrenal steroid production and, at higher doses, also causes accumulation of free cholesterol, resulting in adrenocortical apoptosis [66] and reduced ACTH-stimulated adrenocortical steroid production [66, 67]. In a phase 2 multicenter, single-blind, placebo-controlled, dose-titration study [68], 10 poorly controlled 21OHD patients complemented their supraphysiological glucocorticoid therapy with twice daily 125 mg Nevanimibe for two weeks, followed by a single-blind 2-week placebo washout. Nevanimibe dose was gradually titrated up to 1000 mg/day if the primary outcome measure -being 17OHP within two times the upper limit of normal- was not met. Two subjects met the primary endpoint at a dose of 250 mg or 1000 mg twice daily. Another 5 patients did show a reduction in 17OHP levels between 27 and 72%. However, adverse events were reported. The most commonly reported side effect was gastrointestinal (30% during Nevanimibe and 20% during wash-out placebo period). Nevanimibe failed to reduce androstenedione levels, but this may have been due to the short period of this trial and may indicate the requirement of higher doses [69]. However, a following sixteen-week dose-titration trial (NCT03669549) had been terminated following an interim data review and further investment in Nevanimibe had been discontinued.

Abiraterone acetate (Janssen research & development, Raritan, NJ, USA) is a potent and selective inhibitor of the enzyme CYP17A1 (17α-hydroxylase/17,20-lyase), commonly used in the treatment of prostate cancer [70]. In a phase 1 dose-escalation study, six women with 21OHD treated with physiological doses (20 mg/day) of HC co-administered 100 or 250 mg abiraterone acetate once-daily for six days [71]. When receiving 100 mg/day, serum mean androstenedione normalized in only three women, which prompted a second treatment period at a higher dose. When receiving abiraterone acetate at 250 mg/day, which is approximately 5–15% of the dose equivalent used to treat prostate cancer, mean androstenedione levels normalized in all six women. No severe adverse events occurred, and treatment was generally well tolerated. Next to androstenedione and testosterone, also the 11-oxygenated androgens decreased markedly after 6 days of abiraterone treatment from baseline [72]. While abiraterone treatment may allow for physiological glucocorticoid doses to normalize androgen production, attenuation of the negative feedback loop by reduced glucocorticoid doses may be associated with elevated ACTH levels, potentially predisposing male patients to TART development. A phase 1 study on abiraterone in pre-pubescent children with 21OHD is expected to finish soon (NCT02574910) and a longer-term (2-year) phase 2 randomized placebo-controlled trial on abiraterone in pre-pubescent children is intended (NCT03548246). As CYP17A1 is an important enzyme in the production of gonadal hormones it can decrease endogenous sex hormone production and is therefore not suitable for long term treatment in adults.

Mitotane is a selectively adrenotoxic drug that inhibits ACTH-induced adrenal steroidogenesis [73, 74]. It acts as an inhibitor of CYP11A1, CYP11B1, and CYP11B2, but also has direct toxic effects on the adrenal gland. While mitotane is the primary treatment option for adrenocortical carcinoma [75], it is not recommended as routine therapy for CAH, because of its potential teratogenicity and significant toxicities [2]. Mitotane has been successfully used to restore fertility in men with 21OHD and TART, for men whose TART did not respond to increasing glucocorticoid doses [76, 77]. TART size reduced or TART completely disappeared in four of six reported patients [76]. Mitotane enhances HC inactivation by induction of CYP3A4 [78]. Therefore, while it may have potential to reduce androgen excess, it has no potential to lower glucocorticoid doses, but rather requires increased glucocorticoid dosing [77, 78], which should be closely monitored to prevent the occurrence of adrenal crises [77].

To definitively eliminate adrenal androgens, adrenalectomy has been suggested as an effective tool, especially in patients with longstanding poor hormonal control. However, major concerns have been raised [79]. First, bilateral adrenalectomy leads to a complete loss of cortisol and adrenal precursor steroids, making the patient more vulnerable to adrenal crisis especially in patients with known poor compliance. Second, the complete lack of cortisol leads to increased ACTH levels with consequently a higher risk to develop Nelson’s disease, a well-known complication in ACTH-dependent Cushing disease. Finally, due to the chronically exposure of high levels of ACTH, adrenal rests may develop in the testes of male patients (TART) or in female patients [80, 81]. Therefore, bilateral adrenalectomy is generally not recommended [2].

### Targeting androgen function

Elevated estrogens, aromatized from adrenal androgens, are the main cause of advanced skeletal maturation in childhood leading to impaired final height [82]. Administration of an aromatase inhibitor can be beneficial to reduce bone age advancement in children with CAH [83, 84], but is regretfully associated with higher androgen levels. Therefore, co-administration of both an aromatase inhibitor and an anti-androgenic drug (avoiding signs of hyperandrogenism) in addition to the routinely used fludrocortisone and HC is a potential approach to achieve normal growth and development in 21OHD children with severely advanced bone age due to negative consequences of previous suboptimal treatment. This four-drug combination therapy has been found effective in controlling growth rate and bone maturation in children with CAH [85, 86]. Normal linear growth and bone maturation during 2 years of follow up was noted and no severe adverse effects were reported [85, 86]. However, final height data and long-term follow up data are still lacking. Another clinical study assessing the antiandrogenic capacity of flutamide in combination with the aromatase inhibitor letrozole was discontinued in two of eight children due to flutamide-induced side-effects [87]. A long-term phase 2 study, assessing if this combination therapy can safely maintain normal growth and development throughout childhood and adolescence (until adult height is reached), is ongoing and is expected to finish at the end of 2022 (NCT00001521). While this four-drug treatment regimen allows for lower physiological HC doses, this may be partially due to the negative effect of flutamide on cortisol clearance [88]. Nonetheless, when reducing HC dose, potential TART development should be closely monitored because of potentially elevated ACTH levels, as a consequence of reduced negative feedback by HC [85, 86], and glucocorticoid dose should be increased if testicular nodules are detected [86]. Because of the potential hepatotoxic effect, side-effects need to be monitored closely and it is currently not recommended to use aromatase inhibitors in combination with an anti-androgen outside a clinical trial setting [86].

### Gene-based therapy

D﻿espite the ﻿development of improved glucocorticoid formulations and additional therapies that may improve glucocorticoid dosages to physiological levels, therapy remains limited by its unresponsiveness and ability to adapt to the physiological demand. Gene-based therapy anticipates restoring the HPA-axis regulated glucocorticoid and mineralocorticoid production by replacement of the functional *CYP21A2* gene with consequently 21-hydroxylase production. Preclinical studies have demonstrated promising results; a single intra-adrenal injection or less invasive intra-muscular injection of an adenoviral vector encoding *CYP21A2/CYP21A1* in 21OHD mice successfully induced *CYP21* expression and resulted in corticosterone production at similar levels as observed in wild-type mice [89] or a decline in progesterone (precursor upstream *CYP21* defect) to *CYP21*-metabolized deoxycorticosterone ratio [90]*.* Adeno-associated virus (AAV) has emerged as leading vehicle for gene therapy [91], with the potential for more sustained expression. Intravenous AAV-mediated human *CYP21A2* vector transfer resulted in 21-hydroxylase protein expression in the adrenals of 21OHD mice, albeit at lower levels than endogenous 21-hydroxylase levels in control mice. Still, following injection, progesterone level decreased by 42% and remained near normal in 21OHD mice until the end of the study (15 weeks) [92]. Another study reported therapeutic efficacy, in terms of 21-hydroxylase protein expression and normalized progesterone and ACTH production, for a period of 8 weeks [93]. The authors stated that because of the continuous renewal of the adrenal cortex, AAV-mediated gene therapy has a limited duration of therapeutic efficacy. Nonetheless, assessment of the durability of AAV based gene therapy in cynomolgus monkeys demonstrated durable detection of vector genome copies up to 24 weeks (preliminary non-peer reviewed results [94]). A phase 1/2, first-in-human, open-label, dose-escalation study is designed to study the safety, tolerability, and efficacy of AAV-based gene therapy in adult 21OHD patients (NCT04783181).

### Cell-based therapy

Another therapeutic strategy to restore HPA-axis-regulated glucocorticoid and mineralocorticoid production is the transplantation of adrenocortical(-like) cells that are either isolated from animals or human donors or generated *de novo*. Bovine adrenocortical cells encapsulated in either alginate or an immune-isolating device have been successfully transplanted into bilaterally adrenalectomized immunocompetent rats. The adrenocortical transplants remained ACTH-responsive, produced cortisol, and rescued the animals from the lethal effect of adrenalectomy, without the need of immunosuppressants [95]. Alternatively, several studies have shown the possibility to generate adrenal-like steroidogenic cells from cells isolated form mice or human sources [96, 97]. Cells can be reprogramed to a steroidogenic phenotype through overexpression steroidogenic-factor 1 (SF1) and treatment with cAMP [96]. Easily accessible cells from human blood, urine, or skin have been successfully reprogrammed into steroidogenic cells [97] by forced expression of SF1, activation of the protein kinase A pathway, and the presence of luteinizing-hormone releasing hormone (LHRH). Reprogramming resulted in *de novo* expression of steroidogenic enzymes and secretion of cortisol in a stimulus-dependent manner. Cells were viable when transplanted into the mouse adrenal gland tissue. Interestingly, lentiviral delivery of the wild-type *CYP21A2* gene to CAH patients’ obtained and induced steroidogenic cells rescued cortisol secretion and lowered testosterone secretion *in vitro* [97]. Only limited *in* *vivo* studies have been performed using reprogrammed adrenal-like steroidogenic cells [98]. Long-term viability as well as the ability of these induced steroidogenic cells -potentially after correction of the enzymatic defect- to restore glucocorticoid and mineralocorticoid levels to physiological levels after transplantation in animal models of CAH need to be studied but seem to hold great promise.

## Summary

It is well recognized that current 21OHD treatment is suboptimal and potentiates the development of severe long-term complications. Clinicians have to deal with the effects of elevated adrenal androgens in untreated or poorly treated patients, and with the effects of cortisol in overtreated patients, or both. Until gene therapy or adrenocortical cell transplantation becomes feasible to restore HPA-axis regulated adrenal steroid production, daily glucocorticoid replacement therapy remains indispensable. The main challenge is the suppression of adrenal androgens produced by the chronically stimulated adrenal cortex. The use of modified release medication is a promising step to more effectively inhibit the HPA-axis that, thereby, may allow the use of lower physiological glucocorticoid doses. In the future, co-administration of CRF1 receptor antagonists, ACTH antibodies, or ACTH receptor antagonists may improve biochemical control and/or allow for further reduction of glucocorticoid dose.

## Abbreviations

CRH: Corticotropin-releasing hormone
ACTH: Adrenocorticotropic hormone
GC: Glucocorticoid
CSHI: Continuous subcutaneous hydrocortisone infusion
Preg: Pregnenolone
Prog: Progesterone
17OHPreg: 17-Hydroxypregnenolone
17OHP: 17-Hydroxyprogesterone
DHEA: Dehydroepiandrosterone
A4: Androstenedione
T: Testosterone
11OHA4: 11β-Hydroxyandrostenedione
11KA4: 11-Ketoandrostenedione
11OHT: 11β-Hydroxytestosterone
11KT: 11-Ketotestosterone
CYP17A1: 17α-Hydroxylase/17,20-lyase
CYP21A2: 21-Hydroxlase
CYP19A1: Aromatase
HC: Hydrocortisone
CAH: Congenital adrenal hyperplasia
21OHD: 21-Hydroxylase deficiency
HPA: Hypothalamic-pituitary adrenal
